# Efficacy of Sodium Valproate in the Treatment of Photosensitive Epilepsy (PSE) and the Probable Reasons for the Persistence of Occipital Spikes

**DOI:** 10.1100/tsw.2004.108

**Published:** 2004-07-15

**Authors:** Ebere C. Anyanwu, John E. Ehiri, Andrew W. Campbell

**Affiliations:** ^1^ Neurosciences Research, Cahers Inc., 8787 Shenandoah Park Drive, Suite 122, Conroe, TX 77385, USA; ^2^ Department of Child and Maternal Health, University of Alabama, Birmingham, USA; ^3^ Medical Center for Immune and Toxic Disorders, Spring, TX, USA

**Keywords:** photosensitive epilepsy, sodium valproate, occipital spikes

## Abstract

Intermittent photic stimulation (IPS) in patients with photosensitive epilepsy (PSE) leads to EEG abnormalities, which include generalized discharges with spike and wave activity. This paper investigates 33 PSE patients, 14 (42%) males and 19 (58%) females. The age range was between 8 and 45 years. After the treatment of the patients with sodium valproate (VPA), the EEG examinations showed that the generalized discharges disappeared, while the occipital spikes persisted. The mechanism of action of VPA was re-evaluated in order to ascertain whether or not the persistent occipital was due to a failure in inhibitory postsynaptic potential (IPSP). It was concluded that the possible causes of VPA's inefficacy in abolishing occipital spikes in PSE was not necessarily due to a failure in IPSP, but rather it could be due to a time-dependent failure of certain cells of the visual system to respond positively to the VPA's modulatory activity, probably involving the ionic channels, neurotransmitters, and the second messenger systems. The relationship between occipital spikes and visual evoked response is discussed. The extent to which metabolic processes and neurotransmitters are involved is also evaluated.

## INTRODUCTION

In photosensitive epilepsy (PSE), intermittent photic stimulation (IPS) leads almost invariably to EEG abnormalities, the most common of which is photoparoxysmal response (PPR), i.e., the patients show some generalized discharges with spike and wave activity, 3 Hz, polyspikes, and irregular spike and wave discharges, which are not time locked to the stimulus[1,2,3]. Earlier papers have reported the predominance of occipital spikes in PSE. Naquet et al.[4] observed biphasic spikes or spike and wave discharges in the parieto-temporal areas of 12 patients on photic stimulation, with clinical episodes produced in 7 patients during the discharge period. In 6 of 7 patients, IPS produced generalized spike and wave activity on a repeat stimulation. Fischer-Williams et al.[5] described a woman who showed irregular spike with spike and wave discharges at the occipito-parieto-temporal regions, which varied from side to side with experienced visual abnormalities.

Hishikawa et al.[6] reported that 8 out of 15 (53%) of PSE patients showed an occipital origin for the EEG discharges evoked by photic stimulation. Panayiotopoulos et al.[7] reported that out of 38 patients who showed PPR during IPS, 33 (87%) showed occipital spikes preceding the generalized photoparoxysmal discharge. Although the focal or occipital spikes (which are not related to the flash stimulus) have been described, the generalized slow activity and posterior spikes are not universally accepted as a component of PPR, but the significance of these discharges increases if they continue after the photic stimulation is discontinued.

Gastaut et al.[8] investigated the clinical significance of slow wave discharges in the parieto-occipital regions and found that out of 4,000 patients and 500 allegedly normal volunteers, 7.5% of the epileptic patients and 15% of the normal subjects did have slow wave discharges in the parieto-occipital regions of the brain. In addition, of the patients found with a slow wave posterior discharge, only 47% had a history of epilepsy. Hence, this response is not as specific as the generalized spike and wave discharge. In treating PSE with sodium valproate (VPA), it is noted[9,10] that although the generalized seizure disappeared, the amplitude of N2-P2-N3 due to VPA in relation to predrug levels remained larger in comparison to controls.

In this investigation, we shall look into the treatment of PSE with antiepileptic drugs (AEDs) in general and with VPA in particular. The persistence and the distribution of occipital spikes in PSE after treatment with VPA will be verified. The possible biochemical significance of occipital spikes in PSE will be surveyed and supported by recent research findings.

## MATERIAL AND METHODS

Occipital spikes in a total of 33 PSE patients who attended the Neurophysiology Clinic were studied, out of which 14 (42%) were males and 19 (58%) were females (M/F ratio of 1:1.14). All the patients were treated with VPA, although some had subsequent AED treatment because the previous drugs were ineffective. The age range was between 8 and 45 years. All the patients had a basic EEG including hyperventilation recorded on 16-channel machine using the International 10-20 electrode placement system. A parasagittal montage was used during photic stimulation provided by a Grass P22 photostimulator at light intensity 2 (1,363 cd/m2) with a pattern grid using the 2-sec timer if required[3]. The patients were tested with their eyes either open or closed. The room light standard was artificial light only. The lamp with grid was placed 30 cm from the face of the patients and they were asked to fixate in the center of the lamp, which subtended a visual angle of $25^{o}$. Pattern sensitivity was carried out on the patients with T221 Gratings Generator and Hitachi Monitor at a contrast 90%. Monocular protection was also tested.

## RESULTS

All the PSE patients showed occipital origin for EEG spikes preceding a generalized PPR; 5 (15%) showed occipital spikes in the right occipital region; 3 (9%) in the left occipital region; 19 (58%) in both sides of the occipital regions, while only 3 (9%) in the fronto-occipital regions. Two (6%) of the patients presented occipital spikes in the mid-occipito-temporal region. One (3%) patient had ill-defined occipital spikes, which involved all the regions. On a repeat EEG test after treatment with VPA, the generalized spikes were abolished in all the patients, while the occipital spikes persisted.

Occipital spikes occurred mostly in children between the ages of 5 and 15. Increase in age appears to be associated with significant reduction in the levels of occipital spikes. This finding suggests that age may serve independently as a factor in the occurrence of occipital spikes in PSE. However, some studies[11,12] showed that there was neither a significant relationship between ages, nor a significant relationship to female and male ratios and the success of treatment with VPA. It was also shown that the success of treatment or control of photosensitivity with VPA was dependent on the dose level[13]. But there was no evidence to show that VPA, in controlling PPR, also controlled the occipital spikes. In all the patients, occipital spikes were observed between 7 and 10 flashes per second (fps), some of which appeared either alone or before a PPR. IPS evoked occipital spikes at higher flash rates above 11 and not more than 58 fps in 33 (67%) of the patients. The flash rate of photic stimulation was not determined in 8 (23%), because they were not tested. Pattern stimulation with T221 Gratings Generator and Hitachi Monitor at 90% contrast evoked occipital spikes in 14 (42%) preceding the generalized PPR in the same region as was observed in photic stimulation. Eighteen (55%) patients were not pattern-tested according to our protocol[3] and, therefore, the occipital spike parameters were not known. One of the patients was responsive only to a blank screen.

## DISCUSSION

The AEDs generally used for controlling epileptic seizures are ethoximide, benzodiazepines (chlodiazepoxide, diazepam, oxazepam, lorazepam, medazepam, flurazepam, chlozepate, clonazepam, etc.), barbiturates (phenobarbitone, primodone), VPA, phenytoin, and ACTH, but none of these drugs are effective in the treatment of PSE except VPA, which has become the drug of choice[10,13].

However, since one of the main EEG characteristics of PSE is the prevalence of occipital spikes, it then follows that VPA has the efficacy of abolishing all the characteristics PSE except the occipital spikes. Jeavons (1983) [11] studied more than 500 patients for 13 years and reported that all the patients showed PPR after IPS. They noted that although the patients were receiving a variety of AEDs, there was little evidence to show that such therapy had any effect on the PPR[3,7,11] except VPA. Ethoximide is ineffective in PSE unless the EEG shows 3 cps spike and wave discharges, but effective in controlling absences and myoclonic jerks. Phenobarbitone and, in fact, all barbiturate derivatives (including primodone), invariably have many side effects, which include irritability and difficult behavior in children and depression in adults. All the benzodiazepines have various side effects too[13]. The research findings support the earlier reports, which show that patients with PSE have occipital preceding a generalized discharge and that when treated with VPA, the generalized spikes disappear, while the occipital spikes persist. This persistence of occipital spikes has not yet been explained neurobiochemically or in any way. This needs to be clarified in order to seek the appropriate therapy for the abolition of the persistent occipital spikes in PSE. To do this we need to review the VPA action mechanisms in order to understand fully why VPA is not able to abolish the persistent occipital spikes. It is universally accepted that the action mechanisms of VPA in the treatment of PSE include the enhancement of inhibition, reduction in excitation, and the modulation of ionic channels that are fundamental mediators of neuronal excitability.

### Historical Perspectives of VPA

The scientific and medical history of valproic acid is relatively long, compared with other frequently used psychopharmacologic agents. Valproic acid was used as an organic solvent in research laboratories for 8 decades, until the fortuitous observation of action against pentylenetetrazol-induced convulsions in rodents. Early clinical experience emphasized therapy of absence seizures in primary generalized epilepsies. During 2 decades of controlled trials in partial- and generalized-onset seizures and myoclonus, VPA was established as the prototypical broad-spectrum AED. Anecdotal observations in patients with both epilepsy and migraine headaches, who were started on VPA, led to prospective, randomized trials that established antimigraine efficacy. Early observations suggested antimanic actions; more than a decade later, controlled clinical trials established significant efficacy of VPA in mania[14]. Since its first marketing as an AED over 37 years ago in France, VPA is regarded worldwide as one of the most widely used AEDs in the treatment of epileptic seizures in both adults and children and in view of the diverse molecular and cellular events that underlie different seizure types, the combination of several neurochemical and neurophysiological mechanisms in a single drug molecule might explain the broad antiepileptic efficacy of VPA[15,16]. Valproic acid, a branched-chain carboxylic acid, has a broad spectrum of action as an AED. While effective in myoclonus syndromes and absence epilepsy, the drug has efficacy for patients with generalized convulsive and partial seizures as well. Mechanisms of action are similar to other drugs used to treat epilepsy, in that VPA limits sustained repetitive firing by actions on the voltage sensitive sodium channel. However, the drug facilitates the removal of glutamate from synaptic regions by upregulating glial glutamate transporters while prolonging the action of GABA (gamma-aminobutyric acid) by limiting production of inhibitory transmitter transporter proteins[17]. VPA possesses efficacy in the treatment of various epileptic seizures such as absence, myoclonic, and generalized tonic-clonic seizures. It is also effective in the treatment of partial seizures with or without secondary generalization and acutely in status epilepticus[18].

VPA is a major broad-spectrum AED effective against many different types of epileptic seizures. VPA is a first-line drug in the treatment of primary generalized seizures and syndromes, but it is also effective in other seizure and epilepsy types. Also, a limited number of studies on the efficacy and safety of VPA treatment have shown that therapy-resistant people with intellectual disability can benefit from add-on VPA medication[19]. Mehrotra et al.[20] used VPA as monotherapy in 90 cases with epilepsy that had at least one fit per week, irrespective of the type of seizures. The effect of the drug was evaluated on the basis of change in seizure frequency. Serum valproic acid levels were estimated by homogenous enzyme immunoassay. All the patients with absence (5/5) and myoclonic (3/3) seizures and 80% (42/53) of cases with generalized tonic-clonic seizures became seizure free. Six of ten patients with only tonic seizures became seizure free. An average daily dose of 19.6 mg/kg provided a mean valproic acid level of 81.4 µg/ml in all seizure-free patients. No correlation could be established between VPA dose and serum levels. Mild transient side effects were noted. Although reported VPA hepatotoxicity and haematologic abnormality, no such conditions were observed. VPA effectively controlled seizures in a majority of patients with partial seizures. Serum level monitoring helps to establish an optimal dose to keep the patient seizure free and no correlation could be established between side effects and serum levels[20].

VPA has a number of distinct pharmacological effects on the central nervous system. In experimental animals, it showed clear anticonvulsant activity, an observation that led to its major clinical use as an antiepileptic agent, especially in petit mal seizures[21]. The basis for its clinical efficacy might be related to its ability to enhance central GABAergic neurotransmission or perhaps to its inhibition of $Na^+$ channels. Whether each of the distinct therapeutic effects of VPA has the same molecular basis is not known[21]. VPA's pharmacokinetic profile has been studied extensively, mostly within the context of treatment of epilepsy[22]. Dose-limited absorption, nonlinear plasma protein binding, and multiple metabolic pathways of elimination characterize VPA. Pharmacokinetic drug interactions involving VPA result from its susceptibility to the effects of both enzyme induction and inhibition, along with an ability for weak to moderate inhibition of the metabolic elimination of other drugs and it has an extensive record of use across the lifespan and a good record of tolerability[22].

VPA is available in different dosage forms for parenteral and oral use. All available oral formulations are almost completely bioavailable, but they differ in dissolution characteristics and absorption rates, and in particular, sustained-release formulations are available that minimize fluctuations in serum drug concentrations during a dosing interval and can therefore be given once or twice daily[23]. According to Smith's review[22], valproic acid is about 90% bound to plasma proteins and the degree of binding decreases with increasing drug concentration within the clinically occurring range. Valproic acid is extensively metabolized by microsomal glucuronide conjugation, mitochondrial beta-oxidation, and cytochrome P450-dependent omega-, (omega-1)-, and (omega-2)-oxidation. The elimination half-life is in the order of 9 to 18 h, but shorter values (5 to 12 h) are observed in patients comedicated with enzyme-inducing agents such as phenytoin, carbamazepine, and barbiturates. VPA itself is devoid of enzyme-inducing properties, but it has the potential of inhibiting drug metabolism and can increase by this mechanism the plasma concentrations of certain coadministered drugs, including phenobarbital (phenobarbitone), lamotrigine, and zidovudine.

## Mechanisms of Action of VPA

VPA is currently one of the major AEDs with efficacy for the treatment of both generalized and partial seizures in adults and children. Studies from animal models on structure-relationships indicate that the mechanisms leading to hepatotoxicity and teratogenicity are distinct and also differ from the mechanisms of anticonvulsant action of VPA[15]. Due to its wide spectrum of anticonvulsant activity against different seizure types, it has repeatedly been suggested that VPA acts through a combination of several mechanisms[15]. There is substantial evidence that VPA increases GABA synthesis and release and thereby potentiates GABAergic functions in some specific brain regions, such as substantia nigra, thought to be involved in the control of seizure generation and propagation[15]. Summarily, it could be concluded that VPA reduces the release of the epileptogenic amino acid gamma-hydroxybutyric acid, attenuates neuronal excitation induced by NMDA-type glutamate receptors, exerts direct effects on excitable membranes, alters dopaminergic and serotonergic functions, and is metabolized to several pharmacologically active metabolites[15]. Acting to alter the balance of inhibition and excitation through multiple mechanisms is clearly an advantage for VPA and probably contributes to its broad spectrum of clinical effects. Although the GABAergic potentiation and glutamate/NMDA inhibition could be a likely explanation for the anticonvulsant action on focal and generalized convulsive seizures, they do not explain the effect of VPA on nonconvulsive seizures, such as absences. Although it is often proposed that blockade of voltage-dependent sodium currents is an important mechanism of antiepileptic action of VPA, the exact role played by this mechanism of action at therapeutically relevant concentrations in the mammalian brain is not clearly elucidated[15,16]. These mechanisms are further discussed under VPA’s enhancement of inhibition, blockade of GABA uptake into the neurons of Glia, blockade of NMDA-mediated excitation, modulation of ionic channels, and the effects of neurotransmitters and their overall influence on occipital spikes and inhibitory postsynaptic potentials (IPSPs)

### *VPA Enhancement of Inhibition*

One of the accepted mechanisms of VPA drug action in PSE is based on its ability to facilitate inhibition of abnormal excitatory activities. It is believed that VPA does this by potentiating GABAergic inhibition[13]. Recent studies in VPA action mechanisms have demonstrated a number of ways through which inhibition can be achieved. The most direct way is with the drug's capacity to enter the brain and be converted either to GABA or structurally related compounds with GABAA receptor agonist activity, or analogs of other endogenous inhibitory substances such as glycine and taurine[24,25], since such analogs are lipid soluble, are able to cross the blood-brain barrier, and can be converted to GABA or its analogs. Another strategy is the inhibition of the GABA catabolic enzyme, GABA transaminase (GABA-T). This enzyme is known to enhance the availability of GABA in the synapse and the prevention of GABA breakdown to glutamate and succinic semialdehyde. GABA-T inhibitors are believed, at least theoretically, to potentiate the action of the GABA that is released in the synapse without producing generalized inhibitory effects throughout the brain, as do direct acting GABA agonists[24,25].

### *Blockade of GABA Uptake into the Neurons of Glia*

Another possibility is by the blockade of the uptake of GABA into the neuronal or glial cells. This alternative strategy could have been efficient but for many side effects of the blockers and their inability to penetrate the blood-brain barrier. For example, it is believed that GABA uptake blockers should selectively potentiate the inhibitory effects of synaptically released GABA[13]. Although some conventional blockers of GABA uptake, such as nipecotic acid and guavacine, have significant anticonvulsant potency when administered intracerebroventricularly, they are incapable of penetrating the blood-brain barrier. The derivatives of lipophilic nicopecotic acid have similar anticonvulsant efficacy, but were shown to be toxic in human volunteers[13,24,25]. Neurophysiological evidence supports the concepts that VPA acts on postsynaptic elements, probably on the GABA-receptor complex, and that this GABA-receptor complex involves three or more elements that show different characteristics in binding experiments using radioligands[26].

## Blockade of NMDA-Mediated Excitation

The blockade of NMDA-mediated synaptic excitation has been investigated extensively and observations suggest that its blockade synaptically could be an alternative method in potentiating the inhibitory activity of the VPA drug. Many lines of evidence from *in vitro* physiological studies support the concept that NMDA receptors are critical to epileptiform activity and epileptogenesis, since NMDA antagonists are shown to exhibit a broad spectrum of anticonvulsant activity in animal seizure models[24] and are very effective against maximal electroshock seizures. For example, 2-amino-7-phosphonoheptanoic acid (APH) and 2-amino-5-phosphonovaleric acid (AVP) (which are NMDA antagonists[27]), and their unsaturated analogs (longer-acting carboxyethylesters[28,29,30,31,32]), are known to be effective seizure blockers.

## Modulation of Ionic Channels

Modulation of ion channels ($Na^+$, $K^+$, $Ca^{2+}$, $Cl^-$) or diminutive inhibitory approach to ion neuronal or glial excitability towards effective anticonvulsant efficacy is a very attractive strategy. Nevertheless, there is no experimental evidence to date to substantiate the understanding of the mechanisms in the brain underlying the modulatory or diminutive inhibitory action of VPA to the ion channels. For example, it has not been established whether VPA has the capacity to effect the inhibition of the voltage-gated $Na^+$ channels that mediate the upstroke of action potentials. It is an established fact that these ionic channels ($Na^+$, $K^+$, $Ca^{2+}$, $Cl^-$) participate in the neuronal excitability. The activation of $K^+$ channels are known either to hyperpolarize or inhibit the neurons by suppressing the rate of action potential firing by increasing the opposing influence that the $K^+$ currents normally have on depolarizing $Na^+$ currents. A number of anticonvulsant drugs are known to have positive effects in the opening of the $K^+$ channels (except in the pancreatic B-cells), and are quiescent under normal condition[27]. Some authors have shown that VPA is effective against absence seizures and that its antiabsence activity is a consequence of its ability to inhibit the T-type $Ca^{2+}$ channels in the thalmic neurons[28]. This idea is plausible, since T-type $Ca^{2+}$ channels have been shown to mediate the burst firing of thalmic neurons[28,29].

Consequently, a rational approach for the screening of potential antiabsence drugs is to investigate their activity as antagonists of T-type $Ca^{2+}$ channels. As T-type $Ca^{2+}$ channels are probably primed for activation by GABAB receptor-mediated synaptic potentials, an alternative strategy for suppressing thalmic neuron bursting is the blockade of GABAB receptors. In fact, there is evidence that GABA-mediated neurotransmission is critical to absence seizure. Overall, there have been many intensive investigations in recent years based on these four possible strategies of VPA action mechanisms in the therapy of epilepsy and an overwhelming amount of information in many related journals. Unfortunately there is no conclusive experimental evidence about how VPA affects the control of photosensitive epileptic seizures. Several reviews dealing with this topic can be found in Meldrum[30], Turner and Whittle[31], and Kerwin and Tabernet[32]. It has been proposed that VPA can probably affect seizure control through the four mechanisms discussed, however, these proposals need to be elucidated. It is shown that VPA on single neurons causes an enhancement of neuronal activity in some brain areas (hippocampus and hypothalamus) but does not alter the activity in other areas (e.g., the reticular formation). It does, however, potentiate the inhibitory action of GABA, perhaps by an effect on the uptake or GABA degradation, or an effect on the GABA-receptor complex. Animal studies suggest that the most likely mode of action for VPA is GABAergic inhibition, through an action on the synthesis or further metabolism of GABA. Although it could be possible for VPA to penetrate the blood-brain barrier, however, many result findings in this regard are conflicting[33,34,35] and the metabolic processes responsible for connecting the anticonvulsant properties to the GABAergic system are not clear.

### *VPA Action Mechanisms and Neurotransmitters*

A number of neurotransmitters such as glutamate, aspartate, acetylcholine, and glycine have all been investigated during treatment with VPA and it was found that VPA caused a decrease in the whole brain concentration of aspartate[36,37]. There are many lines of evidence in support of the involvement of other neurotransmitters in the action mechanisms of VPA, more especially the monoamines, the conventional neurotransmitters, and the neuropeptides. The involvement of taurine and serotonin [38,39] in the action mechanisms of VPA have been reported. Many conventional neurotransmitters and modulatory neuropeptides are found in and outside the cortical and intracortical circuitry and are known to play major roles in disease states such as PSE[40]. They occur in the afferent fibers to the cortex from the brain stem and basal forebrain sites. The monoamines (noradrenaline, dopamine, serotonin, glutamate, aspartate), acetylcholine, and taurine have all been implicated in PSE[38,39,40,41,42,43]. In order to understand more the reasons why VPA is incapable of abolishing the occipital spikes in PSE, the roles of these neurotransmitters have to be investigated fully.

### *Occipital Spikes and Inhibitory Postsynaptic Potential (IPSP)*

GABA is synthesized from glutamic acid by the enzyme glutamic-acid-carboxylase and the metabolism of GABA makes use of three different enzymes: GABA-transaminase, succinic-semialdehyde-dehydrogenase, and aldehyde-reductase. It is known that synaptically released GABA is not metabolized, but inactivated by reuptake into the neurons and glia[44]. The neurotransmission activities involved in epileptic seizures include neuronal excitability. It is not yet known whether the persistent occipital spikes in PSE after treatment with VPA is a failure in the IPSP or due to some other metabolic phenomena. In the light of the accepted VPA action mechanisms, it is unlikely that the persistent occipital spikes after treatment with VPA is a failure in IPSP, contrary to the earlier belief[45]. It is argued that if the positive visual evoked response (VER) components are produced by IPSPs[46], then the latency similarities between the negative occipital spikes and the positive VER component could be interpreted as a failure of normal inhibitory mechanisms in PSE.

Jeavons and Harding[3] and Jeavons[46] showed some evidence of the relationship between occipital spikes and VER. However, such pieces of evidence are inconclusive and may not be true in the light of earlier studies[47,48] that could not establish any relationship between occipital spikes and VER. For example, they were unable to find a simple relation between occipital spikes and the VER of patients with PSE. This was confirmed in subsequent studies[48] of 16 patients who were examined in detail; the latency of the occipital spikes was compared to the latency of various components (P1, N2, P2, N3) of their VER obtained in response to IPS at 1–4 fps. They found that in 14 of 16 PSE patients there was no relation between the negative occipital spikes and the negative components of their VER. In two patients, the latency of the negative spike appeared to be related to the latency of the negative wave of a triphasic P2 component (which Gastaut et al.[49] referred to as Vb).

## Efficacy and Adverse Side Effects of VPA

The long-term efficacy and adverse-event profiles of sodium VPA in children with newly diagnosed primary generalized or partial epilepsy showed that sodium VPA was effective in achieving high levels of seizure control in both primary generalized seizures and partial seizures with or without generalization.

Adverse events were mostly mild, few necessitating drug withdrawal. Those particularly associated with VPA were weight increase, alopecia, and appetite increase, compared to carbamazepine, which manifests rashes, somnolence, diplopia, and abnormal gait/ataxia[50]. Hence, there seems no doubt that VPA is effective in the treatment of various types of epilepsy and consequently is better tolerated in the shorter term because of the absence of idiosyncratic skin reactions compared to carbamazepine, which initially may have fewer long-term problems and seem less prone to develop weight gain than patients treated with sodium VPA[19,51,52]. The incidence of toxicity associated with the clinical use of VPA is low, but two rare toxic effects, idiosyncratic fatal hepatotoxicity and teratogenicity, necessitate precautions in risk patient populations[15]. Adverse effects include hepatotoxicity that requires informing patients and establishing clinical monitoring plans. Teratogenicity occurs with VPA and requires informing patients and careful monitoring in women during pregnancy[17]. VPA is more effective in generalized onset seizures (generalized tonic-clonic seizures, absence, myoclonus) although there is no evidence from randomized controlled trials to support this belief[53]. Other commonly reported adverse effects of VPA include gastrointestinal disturbances, tremor, and bodyweight gain[22,23]. Other notable adverse effects include encephalopathy symptoms (at times associated with hyperammonaemia), platelet disorders, pancreatitis, liver toxicity (with an overall incidence of 1 in 20,000, but a frequency as high as 1 in 600 or 1 in 800 in high-risk groups such as infants below 2 years of age receiving anticonvulsant polytherapy), and teratogenicity, including a 1–3% risk of neural tube defects. Some studies have also suggested that menstrual disorders and certain clinical, ultrasound, or endocrine manifestations of reproductive system disorders, including polycystic ovary syndrome, may be more common in women treated with VPA than in those treated with other AEDs. However, the precise relevance of the latter findings remains to be evaluated in large, prospective, randomized studies[22,23].

## CONCLUSIONS

The persistent occipital spikes in PSE may be due to the inability of VPA action to achieve maximum penetration of the blood-brain barrier, since it is already known that the action of VPA is limited to certain brain areas. Second, it is possible that VPA is inefficient in modulating the ionic channels, which are the mediators of neuronal excitability, since there is no experimental evidence to demonstrate VPA's modulatory or diminutive inhibitory action on ionic channels. Third, the occipital spikes may be due to a disruption of protein phosphorylation and hence a failure in the regulation of the neuronal function. The brain has extraordinarily active protein phosphorylation systems compared to non-nervous tissues, which involve a number of second messengers. Second messengers include cyclic adenosine monophosphate (cAMP), cyclic guanine monophosphate (cGMP), calcium ions $Ca^{2+}$, and diacylglycerol. Although, the pieces of clinical and experimental evidence in support of the efficacy of VPA in treating PSE are compelling, much remains to be learned at a number of different levels about the mechanisms of action of VPA. It is hoped that with the advances in molecular neurobiology and neuroscience, the mechanisms and the extent to which VPA is efficacious would undoubtedly be better clarified and understood.

## BIOSKETCHES

**Ebere C. Anyanwu, PhD,** is a Clinical Neurophysiologist and Director of Cahers Neurosciences Research, Inc., Conroe, TX. He is also an Adjunct Professor of Anatomy and Physiology at the North Harris and Montgomery Colleges in Houston and The Woodlands, respectively. E-mail: ebereanyanwu@msn.com

**John Ehiri, PhD,** is an Assistant Professor in the Department of Maternal and Child Health, School of Public Health, University of Alabama, Birmingham. E-mail: jehiri@uab.edu.

**Andrew W. Campbell**, **MD**, is the Chief Executive and Medical Director of the Medical Center for Immune and Toxic Disorders, Spring, TX. Hs is also a specialist in neuroimmunotoxicology.
